# Biomarker testing in MCI patients—deciding who to test

**DOI:** 10.1186/s13195-020-00763-7

**Published:** 2021-01-07

**Authors:** Ingrid S. van Maurik, Hanneke F. M. Rhodius-Meester, Charlotte E. Teunissen, Philip Scheltens, Frederik Barkhof, Sebastian Palmqvist, Oskar Hansson, Wiesje M. van der Flier, Johannes Berkhof

**Affiliations:** 1 Alzheimer Center Amsterdam, Department of Neurology, Amsterdam Neuroscience, VU University Medical Center, Vrije Universiteit Amsterdam, Amsterdam UMC, De Boelelaan 1118, 1081 HZ Amsterdam, The Netherlands; 2 Department of Epidemiology and Data Sciences, Vrije Universiteit Amsterdam, Amsterdam UMC, Amsterdam, The Netherlands; 3 Department of Internal Medicine, Geriatric Medicine Section, Vrije Universiteit Amsterdam, Amsterdam UMC, Amsterdam, The Netherlands; 4 Neurochemistry Laboratory, Department of Clinical Chemistry, Amsterdam Neuroscience, Vrije Universiteit Amsterdam, Amsterdam UMC, Amsterdam, The Netherlands; 5 Department of Radiology and Nuclear Medicine, Amsterdam Neuroscience, Vrije Universiteit Amsterdam, Amsterdam UMC, Amsterdam, The Netherlands; 6 Institutes of Neurology and Healthcare Engineering, University College London, London, England; 7 Clinical Memory Research Unit, Department of Clinical Sciences, Malmö, Lund University, Lund, Sweden; 8 Memory Clinic, Skåne University Hospital, Malmö, Sweden

**Keywords:** Biomarkers, Decision support, MCI

## Abstract

**Background:**

We aimed to derive an algorithm to define the optimal proportion of patients with mild cognitive impairment (MCI) in whom cerebrospinal fluid (CSF) testing is of added prognostic value.

**Methods:**

MCI patients were selected from the Amsterdam Dementia Cohort (*n* = 402). Three-year progression probabilities to dementia were predicted using previously published models with and without CSF data (amyloid-beta1-42 (Abeta), phosphorylated tau (p-tau)). We incrementally augmented the proportion of patients undergoing CSF, starting with the 10% patients with prognostic probabilities based on clinical data around the median (percentile 45–55), until all patients received CSF. The optimal proportion was defined as the proportion where the stepwise algorithm showed similar prognostic discrimination (Harrell’s C) and accuracy (three-year Brier scores) compared to CSF testing of all patients. We used the BioFINDER study (*n* = 221) for validation.

**Results:**

The optimal proportion of MCI patients to receive CSF testing selected by the stepwise approach was 50%. CSF testing in only this proportion improved the performance of the model with clinical data only from Harrell’s C = 0.60, Brier = 0.198 (Harrell’s C = 0.61, Brier = 0.197 if the information on magnetic resonance imaging was available) to Harrell’s C = 0.67 and Brier = 0.190, and performed similarly to a model in which all patients received CSF testing. Applying the stepwise approach in the BioFINDER study would again select half of the MCI patients and yielded robust results with respect to prognostic performance.

**Interpretation:**

CSF biomarker testing adds prognostic value in half of the MCI patients. As such, we achieve a CSF saving recommendation while simultaneously retaining optimal prognostic accuracy.

**Supplementary Information:**

The online version contains supplementary material available at 10.1186/s13195-020-00763-7.

## Background

Biomarkers such as amyloid beta1-42 (Abeta) and phosphorylated tau (p-tau) in cerebrospinal fluid (CSF) provide evidence on the neuropathological process underlying a patient’s cognitive decline [1]. Determining the underlying cause of cognitive complaints is particularly useful in the pre-dementia stage of mild cognitive impairment (MCI), as it provides important prognostic information [2]. Appropriate use criteria for the use of CSF biomarkers have been published, aiming to guide clinicians in the use of these biomarkers. In these criteria, longstanding and unexplained MCI is considered an indication for additional biomarker testing [3, 4]. The clinical practice guidelines of the American Association of Neurology (AAN) for MCI are more reluctant and recommend against the use of biomarkers in clinical practice as it is currently unclear how to value additional diagnostic testing in pre-dementia stages [5]. In line with this practice guideline, clinicians tend to implicitly steer against biomarker testing in MCI patients [6], even when multiple studies have shown the prognostic value of CSF biomarkers in MCI on a group level [7, 8]. We think that this suboptimal use of biomarkers in the clinic might be due to the lack of practical cost-efficient tools.

In a former study, we constructed personalized prognostic models that enable estimation of prognosis in terms of dementia conversion for an individual MCI patient, based on available biomarkers [9, 10]. We showed that the use of CSF biomarkers improves prognostic performance over the use of demographic information and magnetic resonance imaging (MRI) information. Nonetheless, biomarker testing is unlikely to contribute to a more accurate prognosis in every MCI patient [11–13]. Here, we took as a starting point the notion that these same models could have additional value as a decision support tool, to aid clinicians in selecting patients for additional CSF biomarker testing.

We aimed to derive an algorithm to select MCI patients for CSF testing and to provide an estimate of the optimal proportion of patients to undergo CSF biomarker testing.

## Methods

### Patients

We selected *n* = 402 patients with a baseline diagnosis of MCI from the Amsterdam Dementia Cohort [14, 15]. Inclusion criteria were availability of MRI data, CSF data and at least 6 months of follow-up. Diagnostic workup consisted of a standardized 1-day baseline assessment. Clinical diagnosis was made by consensus in a multidisciplinary meeting [14]. Until early 2012, the MCI diagnosis was based on Petersen’s criteria [16]. From 2012 onwards, we used the core clinical criteria of the National Institute on Aging-Alzheimer’s Association (NIA-AA) criteria for MCI [2]. Standardized annual follow-up included a follow-up visit with the neurologist and neuropsychologist. The diagnosis was re-evaluated in a multi-disciplinary meeting of the professionals involved. Specific dementia types were diagnosed using established clinical criteria [17–22].

### MRI

Scans before 2008 were performed on 1.0 and 1.5 Tesla scanners (Siemens Magnetom Avanto, Vision, Impact and Sonata, GE Healthcare Signa HDXT). From 2008 and on, MRI of the brain was performed on 3 T scanners (MR750, GE Medical Systems, Milwaukee, WI, USA; Ingenuity TF PET/MR, Philips Medical Systems, Best, The Netherlands; Titan, Toshiba Medical Systems, Japan). All images were performed according to a standardized protocol [23], of which we only used sagittal 3D T1-weighted images with coronal reformats in this study. All scans were reviewed by experienced neuroradiologists. We quantified left and right hippocampal volumes (HCV, mL) using FSL FIRST (FMRIBs Integrated registration and segmentation tool), which were summed for analysis [24].

### CSF analysis

CSF was obtained by lumbar puncture, collected in polypropylene tubes (Sarstedt, Nurmberg, Germany), and processed according to international guidelines [25–27]. Abeta (1-42) and phosphorylated tau (p-tau) concentrations were measured using sandwich ELISAs (Innotest, Fujirebio, Gent, Belgium [28]. We adjusted Abeta concentrations for upward drift [29].

### Stepwise approach

To determine which proportion of MCI patients should receive additional biomarker testing, we applied a stepwise approach. The procedure consisted of three steps.

#### Step 1: obtain progression probability

We took as a starting point our recently published and validated prognostic models to predict probability of progression to dementia within 3 years in MCI patients. These models were constructed with Cox regression and are described and validated in van Maurik et al. (2019) [9]. Here we assigned dementia progression probabilities (range 0–100%) to patients based on clinical data only (i.e. without CSF biomarkers), based on two diagnostic scenarios and using the following two models [9]:

1. Prognostic model based on demographic characteristics only (age, sex, and Mini-Mental State Examination (MMSE) score) further referred to as “demographics only”
2. Prognostic model based on demographic characteristics and hippocampal volume (HCV) (age, sex, MMSE and HCV; further referred to as “demographics and MRI.”

We report Harrell’s C statistics [30] and 3-year Brier scores [31, 32]. Harrell’s C statistic compares event times of pairs of patients and hence is a measure of how well the model discriminates between patients with different times to dementias. A Harrell’s C score does however not mean that the model’s progression probabilities are well-calibrated to the data. Therefore, we report Harrell’s C together with the 3-year Brier score. The 3-year Brier score measures the quadrative distance between the dementia status after 3 years and the model progression probability, thus is reflective of prognostic accuracy capturing both discrimination and calibration.

#### Step 2: refine prognosis using a stepwise approach

We reasoned that patients with high or low progression probabilities based on clinical data only are unlikely to benefit from additional biomarker testing, in terms of improving the prognostic accuracy for dementia conversion. On the other hand, in patients that have an initial progression probability in the center of all patients’ prognostic probabilities, additional biomarker testing could improve the prognosis. Therefore, in our MCI group, we defined the median 3-year progression probability according to the demographic and/or MRI information as most uncertain since it is the predicted prevalence of 3-year progression.

Subsequently, we used a stepwise approach and added additional CSF biomarker data (Abeta and p-tau concentration in CSF; further referred to as additional CSF) to refine prognosis in the 10% (between percentile 45–55) of patients surrounding the median 3-year progression probabilities. Of note, due to the high correlation of p-tau and total tau (t-tau), t-tau concentrations are not included in the models. Details on the selection of variables in the models are described elsewhere [9, 10]. Meaning that after the first 10% of patients, the prognosis is refined with biomarker data in 20% of patients (between percentile 40–60), then 30% (between percentile 35–65), and so on. Supplemental Table 1 provides an overview of 3-year prognostic probabilities (i.e., probability thresholds) that correspond with these percentiles. Patients with 3-year progression probabilities outside these percentile ranges receive a prognosis from the more simple demographic or MRI model. We performed this stepwise approach by fivefold cross-validation and added additional CSF biomarkers on (1) the demographics information only and (2) demographics and MRI. Overall cross-validated performance of this stepwise model was defined based on the combination of the proportion of patients with probabilities based on clinical information only and the proportion of patients with additional CSF biomarker testing.

#### Step 3: classification performance comparison

We plotted cross-validated Harrell’s C and 3-year Brier scores of stepwise models with increasing proportion of patients receiving biomarker testing against the models with clinical data only (demographics only/demographics and MRI) and the model with additional CSF testing for all patients. This allowed us to identify the optimal proportion of patients where the stepwise approach performed better than the model with clinical data only and equally good as the additional CSF biomarker model in terms of prognostic discrimination (Harrell’s C) and prognostic accuracy (3-year Brier scores).

As we used percentiles of the calculated prognostic probabilities with demographic and/or MRI data, the optimal proportion that is selected corresponds with certain demographic or MRI-model derived probabilities (supplemental Table 1). As a result, the optimal proportion also provided us with an algorithm that defines the threshold of demographic or MRI-model derived probabilities where additional biomarker testing would be indicated, further referred to as probability thresholds.

### Evaluation of stepwise approach

Lastly, we applied the identified probability thresholds found by the stepwise approach in the BioFINDER cohort [33]. From the BioFINDER study, we included *n* = 221 patients with a baseline diagnosis of MCI with available MRI and CSF data and at least 6 months of follow-up. Prognostic probabilities are calculated based on demographic information only and on demographic and MRI information. Based on the identified probability thresholds, the prognosis is refined with additional CSF for only a proportion of patients. Discriminative performance and prediction accuracy in this independent cohort was defined on the combination of the proportion of patients with probabilities based on clinical information only and the proportion of patients with additional CSF biomarker testing.

We illustrate the practical use of the developed algorithm with two cases, one in whom additional CSF testing adds prognostic information, and one where it did not add prognostic information. For the reader to appreciate the clinical characteristics of MCI patients that were or were not selected for additional CSF testing, we will report on the clinical and demographic data for selected patients, patients below the lower probability threshold (not selected) and patients above the upper probability threshold (not selected).

## Results

Table 1 presents the patient characteristics. Mean age of the MCI patients was 66 ± 8 years, 164 (41%) were female, and mean MMSE score was 27 ± 2 points. Overall, 189 (47%) patients progressed to dementia during 3 ± 2 years of follow-up.

**Table 1:** Patient characteristics

	Total ***n*** = 402	Non-progressors ***n*** = 213	Progressors ***n*** = 189
Age	66 ± 8	65 ± 8	67 ± 8
Sex, No. F (%)	164 (41%)	76 (36%)	88 (47%)
MMSE	27 ± 2	27 ± 2	26 ± 3
Follow-up	3 ± 2	3 ± 2	3 ± 2
MRI			
HCV (cm^3^, sum)	6.8 ± 1	7.1 ± 1	6.5 ± 1
CSF			
Abeta (1-42) pg/ml	778 (652–1083)	976 (732–1191)	686 (596–783)
t-tau pg/ml	397 (265–626)	291 (207–422)	566 (379–794)
pTau pg/ml	62 (44–85)	51 (36–69)	76 (59–100)

*CSF* cerebrospinal fluid, *HCV* hippocampal volume, *MMSE* Mini-Mental State Examination, *MRI* magnetic resonance imaging. CSF was measured with Innotest and Abeta values are drift corrected [29]. Of note: t-tau is not included in the models

**Fig. 1:**
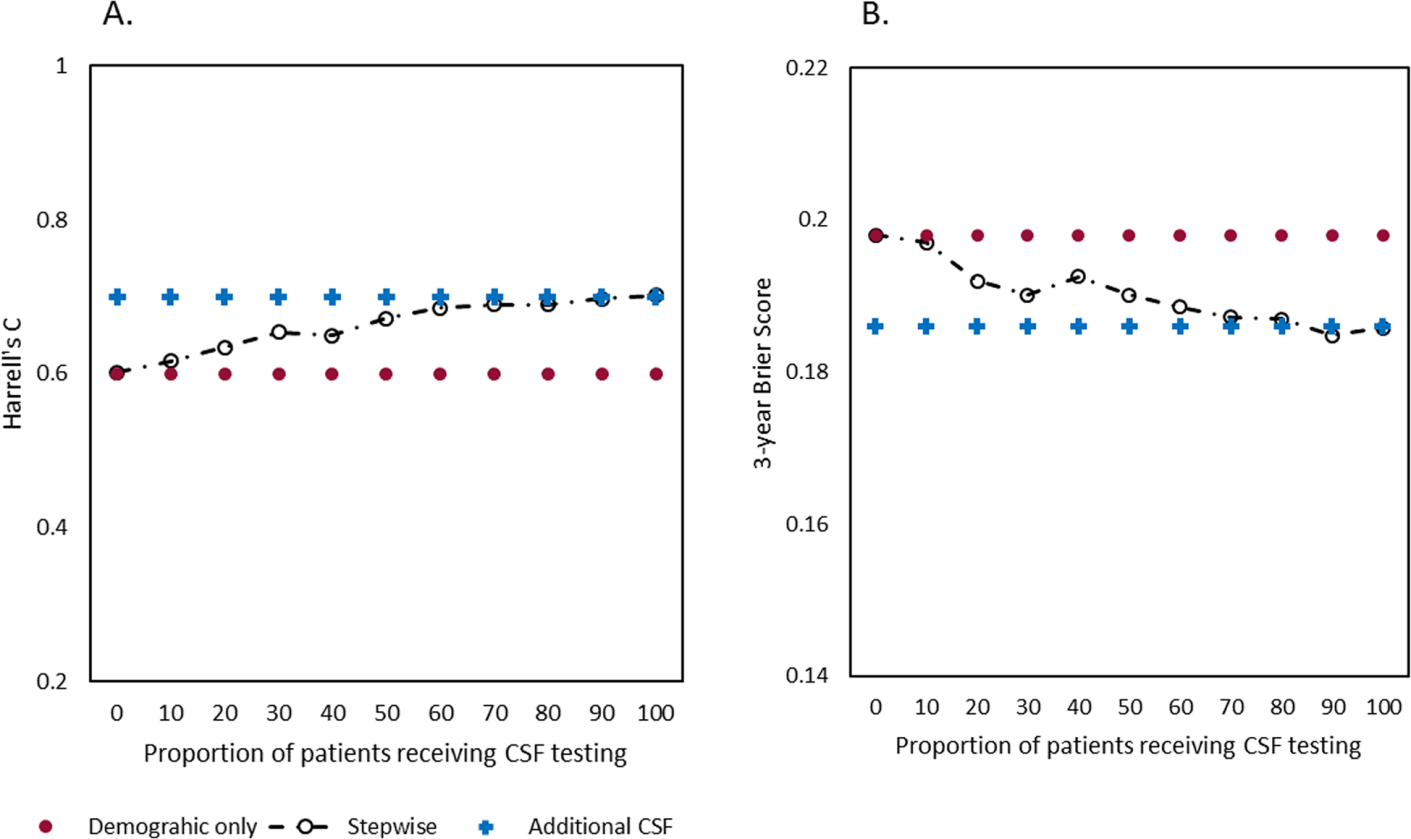
Model performance comparison from demographic only to additional CSF. Comparison of model performance of the stepwise approach (black) from demographic information (red) only to additional CSF testing (blue). **a**. Prognostic discrimination measured with cross-validated Harrell’s C. **b**. Prognostic accuracy measured with cross-validated 3-year brier scores. A lower brier score indicates a better prognostic accuracy. CSF, cerebrospinal fluid

In Fig. 1, the stepwise approach from demographic information only to additional CSF testing is shown. This figure shows the prognostic discrimination and prognostic accuracy of the stepwise model in comparison with demographic information only (Harrell’s C = 0.60, 3-year Brier score = 0.198) and demographics with additional CSF model when CSF results were included from all patients (Harrell’s C = 0.70, 3-year Brier score = 0.186). The discriminative performance of the stepwise model started to increase if 10% of the patients surrounding the median received CSF testing. The discriminative performance of the stepwise model gradually further increased, until it performed similarly to the CSF model (Fig. 1a) when 50% of the patients underwent CSF testing (Harrell’s C = 0.67). Brier scores showed a similar pattern and were comparable with the CSF models if also 50% of the patients received CSF (3-year Brier score = 0.190, Fig. 1b).

**Fig. 2:**
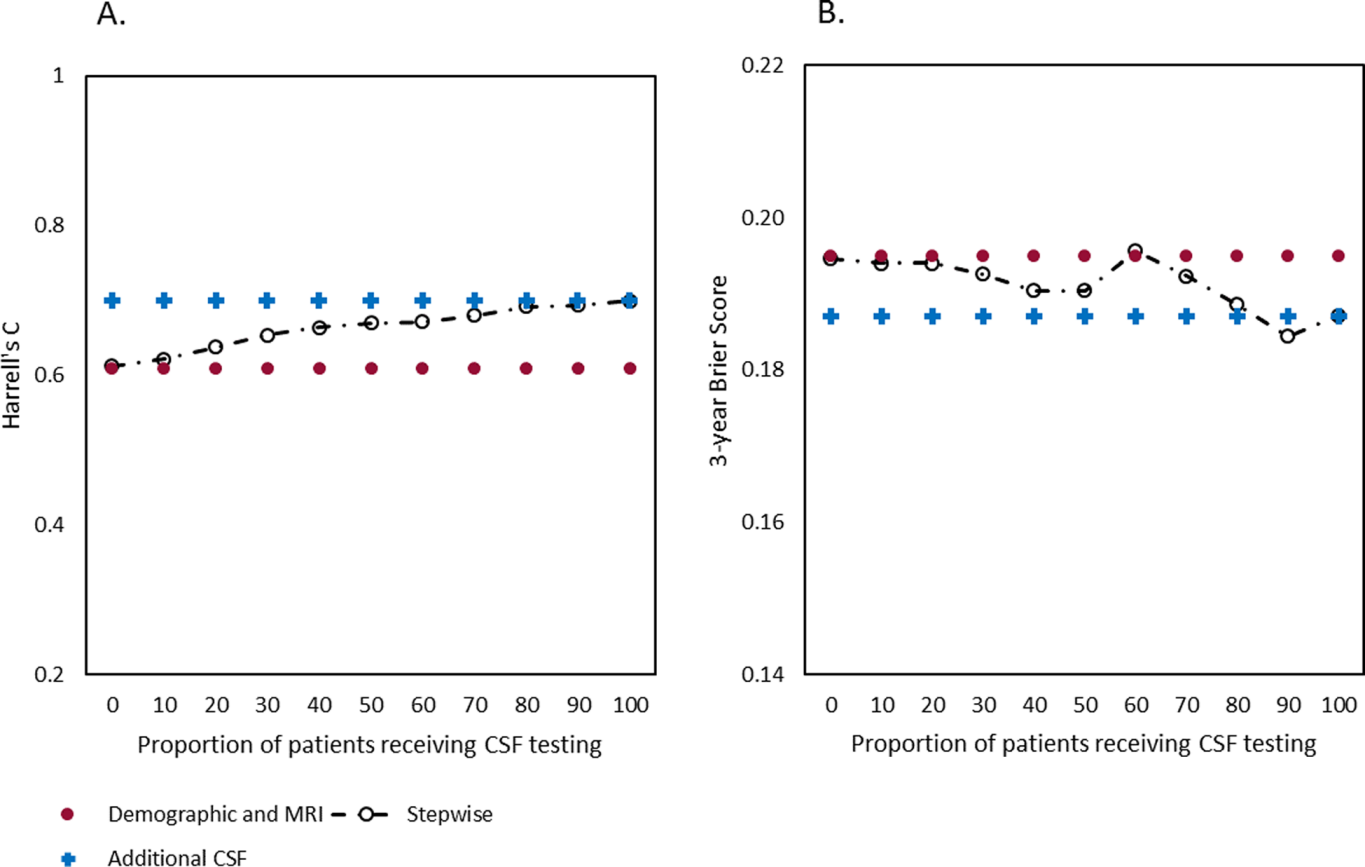
Model performance comparison from demographics and MRI to additional CSF. Comparison of model performance of the stepwise approach (black) from demographic and MRI information (red) only to additional CSF testing (blue). **a**. Prognostic discrimination measured with cross-validated Harrell’s C. **b**. Prognostic accuracy measured with cross-validated 3-year brier scores. A lower brier score indicates a better prognostic accuracy. CSF, cerebrospinal fluid; MRI, magnetic resonance imaging

Figure 2 shows the stepwise approach from demographic and MRI information (Harrell’s C = 0.61, 3-year Brier score = 0.195) to additional CSF testing (Harrell’s C = 0.70, 3-year Brier score = 0.187). The stepwise model again started to increase if 10% of the patients received CSF testing and performed similarly to the CSF in all patients model (Fig. 2a) when 50% of the patients received CSF testing (Harrell’s C = 0.67). Brier scores showed a similar, although more wiggly, pattern and was comparable with the full CSF model if also 50% of the patients received CSF (3-year Brier score = 0.190, Fig. 2b). Table 2 shows the characteristics of patients that were and were not selected based on demographic and/or MRI information.

**Table 2:** Characteristics of MCI patients stratified by selection for additional CSF testing

	Demographics only model			Demographics and MRI model		
	Not selected Low prob. ***N*** = 103	Selected ***N*** = 199	Not selected High prob. ***N*** = 100	Not selected Low prob. ***N*** = 100	Selected ***N*** = 202	Not selected High prob. ***N*** = 100
Age	60 ± 7	66 ± 7	71 ± 7	60 ± 7	66 ± 7	70 ± 7
Sex, No. F (%)	10 (10%)	81 (41%)	73 (73%)	28 (28%)	119 (59%)	47 (47%)
MMSE	28 ± 1	27 ± 2	24 ± 2	28 ± 1	27 ± 2	24 ± 2
MRI						
HCV (cm^3^, sum)	7.3 ± 1	6.8 ± 1	6.3 ± 1	7.6 ± 1	6.8 ± 1	6.0 ± 1
CSF						
Abeta (1-42) pg/ml	995 ± 301	844 ± 278	768 ± 235	976 ± 311	851 ± 276	778 ± 245
t-tau pg/ml	332 ± 192	516 ± 372	564 ± 312	360 ± 234	489 ± 294	585 ± 433
pTau pg/ml	52 ± 24	72 ± 37	77 ± 34	56 ± 26	69 ± 33	79 ± 40

*CSF* cerebrospinal fluid, *HCV* hippocampal volume, *MMSE* Mini-Mental State Examination, *MRI* magnetic resonance imaging. CSF was measured with Innotest and Abeta values are drift corrected [29]. Of note: t-tau is not included in the models

Subsequently, we evaluated the identified probability thresholds in the BioFINDER study (supplemental Table 1). Patient characteristics of the BioFINDER study are reported in supplemental Table 2. Applying the identified probability thresholds by the stepwise approach in the BioFINDER study would select 51% for CSF biomarker testing based on demographic information. Based on demographic and MRI information, the algorithm would select 48% of patients for additional CSF testing. CSF testing only in this proportion of patients yielded a better performance in comparison with CSF testing in none of the patients and a similar performance in comparison with CSF testing in all MCI patients (supplemental Table 3).

To illustrate the practical implementation of our algorithm for additional CSF biomarker testing, we present two clinical cases. For patient A, based on age (70 years), sex (female), and MMSE score (28), the 3-year progression probability was estimated to be 49.7%. This probability falls within the identified probability of the 50% of patients surrounding the median, and therefore additional CSF testing would be recommended. Adding CSF information (Abeta = 1188, p-tau = 47) resulted in a far lower progression probability of 17.8%.

For patient B, both demographic and imaging information were available. Based on age (54 years), sex (male), MMSE (29), and HCV (sum; 7 cm^3^), the 3-year progression probability was estimated to be 14.0%. This probability falls outside the identified probabilities of the 50% of patients surrounding the median based on demographic information and MRI. As the progression probability was already low, the algorithm does not recommend to add CSF testing. The progression probability of the ATN model (additional CSF testing; Abeta = 1349, p-tau = 44) for this patient was 9.2% and showed that CSF indeed did not meaningfully alter the estimated prognosis of this patient.

## Discussion

We developed an algorithm to identify those MCI patients most likely to benefit from additional biomarker testing. We showed that CSF biomarker testing adds prognostic value to clinical information in half of the MCI patients. The findings were replicated in an independent cohort. As such, we achieved a CSF saving recommendation without reducing prognostic accuracy.

In the decision to perform additional diagnostic testing, it is important to specify to what end a diagnostic test is performed, e.g., to identify or exclude Alzheimer’s disease (AD) pathology, predict clinical progression, change disease management, and/or improve well-being. The BIOMARKAPD project, a multidisciplinary working group, ranked these clinical questions on importance, and showed that CSF biomarkers are particularly useful to identify AD pathology and to predict progression to AD dementia in MCI patients [34]. Their recommendations are similar to those of the appropriate use criteria for CSF [4]. Both advise on CSF testing in all MCI patients. However, these recommendations are based on studies that investigated the additional value of CSF in terms of diagnostic or prognostic accuracy on a group level. In such studies, CSF is tested in all (MCI) patients and provides no information on the usefulness in specific patients. Moreover, the appropriate use criteria fairly state that a comprehensive clinical evaluation should precede the use of CSF biomarkers [4]. Clinicians should then determine, based on the available information, in which patients’ CSF biomarkers contribute to the diagnosis and clinical decision making. Such statements in the appropriate use criteria, however, are hard to operationalize for clinicians, especially in pre-dementia stages.

The current study provides clinicians with an easy-to-use algorithm that uses readily available information (i.e., age, sex, MMSE, and hippocampal volume if available) to identify MCI patients for CSF biomarker measurement. We took as a starting point progression probabilities based on basic clinical information only. By identifying the range of progression probabilities close to the progression prevalence in the population, where CSF is likely to add prognostic value, we allow the clinician to make an informed decision on performing biomarker testing. The clinician could also use this information to inform the patient before embarking on biomarker testing and manage expectations about potential outcomes. The communication of considerations to perform or not perform a diagnostic test was given high priority in a recent Delphi consensus study among clinicians, patients, and caregivers [35]. The BIOMAKAPD workgroup also acknowledges the importance of these considerations; they recommend that “in the case of positive biomarkers a personal follow-up plan should be offered and appropriate support should be initiated in the case of symptom progression”. And “in the case of negative AD biomarkers, an intensive follow-up plan may not be necessary”. Although this mentions implications for both possible outcomes, it is still in general terms and does not take available clinical information into account.

In the search for practical guidelines on which patient to test, several previous studies developed prediction models for amyloid positivity. Although these studies differ in their methodological details, they all focus on only one of the pathological hallmarks of Alzheimer’s disease as tauopathy and neurodegeneration are not considered [36, 37]. Moreover, most of these studies compare patients with AD dementia with controls and cannot be generalized to the MCI population. Finally, these algorithms identify individuals most likely to benefit from additional testing to identify amyloid positivity—most relevant in a trial setting, while in clinical settings, the clinical outcome, i.e., progression to (any type of) dementia is more relevant. One previous study used a computer algorithm to select patients in whom CSF testing was likely to contribute to a more accurate differential diagnosis for different types of dementia [38]. In this study, CSF testing was recommended in 26% of the cases. However, MCI patients were not included in this study. In the current study, we extended on the available literature with a keen eye for the needs in clinical practice by providing an algorithm to select MCI patients in whom CSF testing is most likely to contribute to a more accurate prognosis.

One of the strengths of the current study is that our algorithms make use of validated prognostic models to estimate the prognosis of each patient using available clinical information (patient characteristics and/or hippocampal atrophy). Moreover, we used measures that are easily available to the clinician, i.e., patient characteristics, the widely used MMSE score, and hippocampal volume. Although we described this stepwise approach for the decision to perform CSF testing in MCI patients, our approach has general applicability to investigate a stepwise approach from any two prognostic models. The novelty in our study is that we used a data-driven approach to define the proportion of patients that would benefit from additional biomarker testing, i.e., the performance of the stepwise approach should be significantly better than the clinical model and similar to the full (demographics, MRI and CSF) model. Similar approaches have been proposed for the classification of cancer samples by means of high-dimensional genomic markers [39] With our full model we have a measure for amyloid (A), tauopahty (T), and neurodegeneration (N) and thus align with the ATN criteria reported by the NIA-AA [1]. Lastly, we validated our stepwise approach in an independent cohort. The success of this validation may have resulted from the fact that BioFINDER patients had a similar risk profile compared to the MCI patients from Amsterdam, as similar diagnostic guidelines for MCI were used in both cohorts. The usefulness of this stepwise approach in a population with a different composition of risk profiles should be a topic for further research.

### Limitations

Among the limitations is that we were unable to construct a stepwise approach from the demographic model to the MRI model, as the demographic and MRI model performed similarly in our sample (data not shown). Although the addition of MRI does not result in a more accurate prognosis in MCI patients, performing MRI or CT is still valuable to exclude other (reversible) causes for cognitive impairment. Other diagnostic tests, like amyloid-PET, were not part of the current study. In a future study, we aim to apply the same approach to amyloid-PET. Based on previous research on prognostic models in amyloid-PET, we expect similar results as reported here [40]. Another direction for further research is the definition of the prognostic accuracy measure. In this paper, we have chosen two well-established measures of model performance, i.e., Harrell’s C and the Brier score. Harrell’s C has the limitation that it is only a discriminative measure which may select models that are poorly calibrated to the actual data. The Brier score is also a measure of calibration, but the quadratic distance used for measuring accuracy may not be the most appropriate measure in clinical decision making.

## Conclusion

In conclusion, we showed that by performing CSF testing in 50% of the MCI patients the same prognostic accuracy is reached compared to testing all patients. Our algorithm uses prognostic models without CSF data to identify those patients most likely to benefit from CSF testing. This has important implications with respect to cost-efficient use of CSF testing. Furthermore, this approach also aids clinicians to set appropriate expectations before diagnostic testing.

## Supplementary Information

**Additional file 1: Supplemental Table 1.** Probability thresholds for proportion of patients receiving additional CSF testing. **Supplemental Table 2.** BioFINDER patient characteristics. **Supplemental Table 3.** Prognostic discrimination and prognostic accuracy in the BioFINDER study.

## Data Availability

The datasets used and/or analyzed during the current study are available from the Amsterdam Dementia Cohort, via the corresponding author, on reasonable request.

## Abbreviations

AAN: American Association of Neurology
Abeta: Amyloid-beta1-42
ATN: Amyloid, tauopathy, neurodegeneration
BioFINDER study: Biomarkers for Identifying Neurodegenerative Disorders Early and Reliably study
BIOMAKAPD project: Biomarkers for Alzheimer’s disease and Parkinson’s disease project
CSF: Cerebrospinal fluid
HCV: Hippocampal volume
MCI: Mild cognitive impairment
MMSE: Mini-Mental State Examination
MRI: Magnetic resonance imaging
NIA-AA: National Institute on Aging-Alzheimer’s Association
p-tau: Phosphorylated tau

## References

[1] Jack CR, Bennett DA, Blennow K (2018). NIA-AA research framework: toward a biological definition of Alzheimer’s disease. Alzheimers Dement.

[2] Albert MS, DeKosky ST, Dickson D (2011). The diagnosis of mild cognitive impairment due to Alzheimer’s disease: recommendations from the National Institute on Aging-Alzheimer’s Association workgroups on diagnostic guidelines for Alzheimer’s disease. Alzheimers Dement.

[3] Johnson KA, Minoshima S, Bohnen NI (2013). Appropriate use criteria for amyloid PET: a report of the Amyloid Imaging Task Force, the Society of Nuclear Medicine and Molecular Imaging, and the Alzheimer’s Association. Alzheimers Dement.

[4] Shaw LM, Arias J, Blennow K (2018). Appropriate use criteria for lumbar puncture and cerebrospinal fluid testing in the diagnosis of Alzheimer’s disease. Alzheimers Dement.

[5] Petersen RC, Lopez O, Armstrong MJ (2018). Practice guideline update summary: mild cognitive impairment: report of the Guideline Development, Dissemination, and Implementation Subcommittee of the American Academy of Neurology. Neurology..

[6] Visser LNC, van Maurik IS, Bouwman FH (2020). Clinicians’ communication with patients receiving a MCI diagnosis: the ABIDE project. PLoS One.

[7] Mattsson N, Lonneborg A, Boccardi M (2017). Clinical validity of cerebrospinal fluid Abeta42, tau, and phospho-tau as biomarkers for Alzheimer’s disease in the context of a structured 5-phase development framework. Neurobiol Aging.

[8] Vos SJ, Verhey F, Frolich L (2015). Prevalence and prognosis of Alzheimer’s disease at the mild cognitive impairment stage. Brain..

[9] van Maurik IS, Vos SJ, Bos I, Bouwman FH, Teunissen CE, Scheltens P, Barkhof F, Frolich L, Kornhuber J, Wiltfang J, Maier W, Peters O, Rüther E, Nobili F, Frisoni GB, Spiru L, Freund-Levi Y, Wallin AK, Hampel H, Soininen H, Tsolaki M, Verhey F, Kłoszewska I, Mecocci P, Vellas B, Lovestone S, Galluzzi S, Herukka SK, Santana I, Baldeiras I, de Mendonça A, Silva D, Chetelat G, Egret S, Palmqvist S, Hansson O, Visser PJ, Berkhof J, van der Flier WM. Alzheimer's Disease Neuroimaging Initiative. Biomarker-based prognosis for people with mild cognitive impairment (ABIDE): a modelling study. Lancet Neurol. 2019;18(11):1034–44. 10.1016/S1474-4422(19)30283-2. 10.1016/S1474-4422(19)30283-2 31526625

[10] van Maurik IS, Zwan MD, Tijms BM (2017). Interpreting biomarker results in individual patients with mild cognitive impairment in the Alzheimer’s biomarkers in daily practice (ABIDE) project. JAMA Neurol.

[11] Babapour Mofrad R, Visser LNC, Fruijtier AD (2019). Cerebrospinal fluid collection: an informative animation video for patients and caregivers. Alzheimers Dement (Amst).

[12] Blennow K, Dubois B, Fagan AM (2015). Clinical utility of cerebrospinal fluid biomarkers in the diagnosis of early Alzheimer’s disease. Alzheimers Dement.

[13] Duits FH, Martinez-Lage P, Paquet C (2016). Performance and complications of lumbar puncture in memory clinics: results of the multicenter lumbar puncture feasibility study. Alzheimers Dement.

[14] van der Flier WM, Pijnenburg YA, Prins N (2014). Optimizing patient care and research: the Amsterdam Dementia Cohort. J Alzheimers Dis.

[15] van der Flier WM, Scheltens P (2018). Amsterdam dementia cohort: performing research to optimize care. J Alzheimers Dis.

[16] Petersen RC, Smith GE, Waring SC (1999). Mild cognitive impairment: clinical characterization and outcome. Arch Neurol.

[17] Dubois B, Feldman HH, Jacova C (2007). Research criteria for the diagnosis of Alzheimer’s disease: revising the NINCDS-ADRDA criteria. Lancet Neurol.

[18] McKeith IG, Dickson DW, Lowe J (2005). Diagnosis and management of dementia with Lewy bodies: third report of the DLB Consortium. Neurology..

[19] McKhann G, Drachman D, Folstein M (1984). Clinical diagnosis of Alzheimer’s disease: report of the NINCDS-ADRDA Work Group under the auspices of Department of Health and Human Services Task Force on Alzheimer’s disease. Neurology..

[20] Rascovsky K, Hodges JR, Knopman D (2011). Sensitivity of revised diagnostic criteria for the behavioural variant of frontotemporal dementia. Brain..

[21] Roman GC, Tatemichi TK, Erkinjuntti T (1993). Vascular dementia: diagnostic criteria for research studies. Report of the NINDS-AIREN International Workshop. Neurology..

[22] Neary D, Snowden JS, Gustafson L (1998). Frontotemporal lobar degeneration: a consensus on clinical diagnostic criteria. Neurology..

[23] Wattjes MP, Henneman WJ, van der Flier WM (2009). Diagnostic imaging of patients in a memory clinic: comparison of MR imaging and 64-detector row CT. Radiology..

[24] Patenaude B, Smith SM, Kennedy DN, Jenkinson M (2011). A Bayesian model of shape and appearance for subcortical brain segmentation. Neuroimage..

[25] Duits FH, Teunissen CE, Bouwman FH (2014). The cerebrospinal fluid “Alzheimer profile”: easily said, but what does it mean?. Alzheimers Dement.

[26] Mulder C, Verwey NA, van der Flier WM (2010). Amyloid-beta(1-42), total tau, and phosphorylated tau as cerebrospinal fluid biomarkers for the diagnosis of Alzheimer disease. Clin Chem.

[27] Teunissen CE, Petzold A, Bennett JL (2009). A consensus protocol for the standardization of cerebrospinal fluid collection and biobanking. Neurology..

[28] Duits FH, Prins ND, Lemstra AW (2015). Diagnostic impact of CSF biomarkers for Alzheimer’s disease in a tertiary memory clinic. Alzheimers Dement.

[29] Tijms BM, Willemse EAJ, Zwan MD (2018). Unbiased approach to counteract upward drift in cerebrospinal fluid amyloid-beta 1-42 analysis results. Clin Chem.

[30] Harrell FE, Califf RM, Pryor DB, Lee KL, Rosati RA (1982). Evaluating the yield of medical tests. JAMA.

[31] Gerds TA, Schumacher M (2006). Consistent estimation of the expected brier score in general survival models with right-censored event times. Biom J.

[32] Graf E, Schmoor C, Sauerbrei W, Schumacher M (1999). Assessment and comparison of prognostic classification schemes for survival data. Stat Med.

[33] Palmqvist S, Zetterberg H, Mattsson N (2015). Detailed comparison of amyloid PET and CSF biomarkers for identifying early Alzheimer disease. Neurology..

[34] Herukka SK, Simonsen AH, Andreasen N (2017). Recommendations for cerebrospinal fluid Alzheimer’s disease biomarkers in the diagnostic evaluation of mild cognitive impairment. Alzheimers Dement.

[35] Fruijtier AD, Visser LNC, van Maurik IS (2019). ABIDE Delphi study: topics to discuss in diagnostic consultations in memory clinics. Alzheimers Res Ther.

[36] Palmqvist S, Insel PS, Zetterberg H (2019). Accurate risk estimation of beta-amyloid positivity to identify prodromal Alzheimer’s disease: cross-validation study of practical algorithms. Alzheimers Dement.

[37] Petrone PM, Casamitjana A, Falcon C (2019). Prediction of amyloid pathology in cognitively unimpaired individuals using voxel-wise analysis of longitudinal structural brain MRI. Alzheimers Res Ther.

[38] Rhodius-Meester HFM, van Maurik IS, Koikkalainen J (2020). Selection of memory clinic patients for CSF biomarker assessment can be restricted to a quarter of cases by using computerized decision support, without compromising diagnostic accuracy. PLoS One.

[39] Obulkasim A, Meijer GA, van de Wiel MA (2011). Stepwise classification of cancer samples using clinical and molecular data. BMC Bioinformatics.

[40] van Maurik IS, van der Kall LM, de Wilde A (2019). Added value of amyloid PET in individualized risk predictions for MCI patients. Alzheimers Dement (Amst).

