# Supplementary material for: Biomarker testing in MCI patients—deciding who to test
Source: Alzheimers Res Ther. 2021 Jan 7;13:14. doi: 10.1186/s13195-020-00763-7 (PMC7792312; doi:10.1186/s13195-020-00763-7)
Supplement: Supplementary file 1 — Additional file 1: Supplemental Table 1. Probability thresholds for proportion of patients receiving additional CSF testing. Supplemental Table 2. BioFINDER patient characteristics. Supplemental Table 3. Prognostic discrimination and prognostic accuracy in the BioFINDER study. [file 13195_2020_763_MOESM1_ESM.docx]

**Supplemental Data**

**Biomarker testing in MCI patients – deciding who to test**

Ingrid S. van Maurik^1,2^*, Hanneke F.M. Rhodius-Meester^1,3^, Charlotte E. Teunissen^4^, Philip Scheltens^1^, Frederik Barkhof^5,6^, Sebastian Palmqvist^7,8^, Oskar Hansson^7,8^, Wiesje M. van der Flier^1,2^, Johannes Berkhof^2^

Supplemental Table 1. Probability thresholds for proportion of patients receiving additional CSF testing

Supplemental Table 2. BioFINDER patient characteristics

Supplemental Table 3. Prognostic discrimination and prognostic accuracy in the BioFINDER study

|  |  | **Demographics only model** | | **Demographics and MRI model** | |
| --- | --- | --- | --- | --- | --- |
| **Proportion of patients** | **Percentiles** | **Lower limit** | **Upper limit** | **Lower limit** | **Upper limit** |
|  |  |  |  |  |  |
| 10 | 45-55 | 40% | 43% | 25% | 28% |
| 20 | 40-60 | 39% | 45% | 24% | 30% |
| 30 | 35-65 | 37% | 48% | 23% | 32% |
| 40 | 30-70 | 36% | 50% | 22% | 34% |
| 50 | 25-75 | 34% | 52% | 21% | 37% |
| 60 | 20-80 | 32% | 56% | 19% | 40% |
| 70 | 15-85 | 30% | 61% | 18% | 44% |
| 80 | 10-90 | 28% | 66% | 17% | 49% |
| 90 | 5-95 | 24% | 73% | 14% | 58% |

**Supplemental Table 1. Probability thresholds for proportion of patients receiving additional CSF testing**

**Supplemental Table 2. BioFINDER patient characteristics**

|  | **Total**  **n=221** | **Non-progressors**  **n=97** | **Progressors**  **n=124** |
| --- | --- | --- | --- |
| Age | 71±5 | 70±6 | 71±5 |
| Sex, No. F (%) | 93 (42%) | 36 (37%) | 57 (46%) |
| MMSE | 27±2 | 28±2 | 27±2 |
| Follow-up | 2±1 | 3±1 | 2±1 |
| *MRI* |  |  |  |
| HCV (cm3, sum) | 6.7±1 | 7.0±1 | 6.4±1 |
| *CSF* |  |  |  |
| Abeta (1-42) pg/ml | 482 (369-711) | 620 (434-811) | 427 (339-554) |
| Tau pg/ml | 355 (268-490) | 298 (247-398) | 418 (308-565) |
| pTau pg/ml | 56 (43-79) | 49 (40-64) | 69 (47-89) |

CSF=Cerebrospinal fluid, HCV=hippocampal volume, MMSE=mini-mental state examination, MRI=magnetic resonance imaging. CSF was measured with Innotest. BioFINDER CSF analysis used "batch-analyses" after one aliquoting step explaining lower values for Abeta. This is accounted for in the prognostic models.^9^ HCVs were calculated with Freesurfer version 5.3.

**Supplemental Table 3. Prognostic discrimination and prognostic accuracy in the BioFINDER study**

| **Proportion of patients receiving CSF testing** | **Models used** | **Harrell's C** | **3 year brier score** |
| --- | --- | --- | --- |
| 0% | Demographics only model | 0.60 | 0.184 |
| 51% | Stepwise from demographic to additional CSF | 0.66 | 0.175 |
| 100% | Additional CSF model* | 0.70 | 0.168 |
| 0% | Demograhpics and MRI model | 0.65 | 0.184 |
| 48% | Stepwise from Demographics and MRI to additional CSF | 0.69 | 0.162 |
| 100% | Additional CSF model** | 0.72 | 0.163 |

ATN= Amyloid, Tauopathy, Neurodegeneration, CSF= Cerebrospinal fluid, MRI=magnetic resonance imaging. *CSF in addition to demographic information, ** CSF in addition to demographic and MRI information.
